# Variants in *ANRIL* gene correlated with its expression contribute to myocardial infarction risk

**DOI:** 10.18632/oncotarget.14721

**Published:** 2017-01-18

**Authors:** Jie Cheng, Meng-Yun Cai, Yu-Ning Chen, Zhi-Cheng Li, Sai-Sai Tang, Xi-Li Yang, Can Chen, Xinguang Liu, Xing-dong Xiong

**Affiliations:** ^1^ Institute of Aging Research, Guangdong Medical University, Dongguan, P.R.China; ^2^ Department of clinical laboratory, The Affiliated Hospital of Guangdong Medical University, Zhanjiang, P.R.China; ^3^ Guangdong Provincial Key Laboratory of Medical Molecular Diagnostics, Guangdong Medical University, Dongguan, P.R.China; ^4^ Department of Cardiovascular Disease, The First People's Hospital of Foshan, Foshan, P.R.China; ^5^ Department of Cardiovascular Disease, The Affiliated Hospital of Guangdong Medical University, Zhanjiang, P.R.China; ^6^ Institute of Biochemistry & Molecular Biology, Guangdong Medical University, Zhanjiang, P.R.China

**Keywords:** ANRIL, single nucleotide polymorphism, myocardial infarction, risk, Gerotarget

## Abstract

*ANRIL* (antisense non-coding RNA in the *INK4* locus), located at the 9p21.3 locus, has been known to be closely associated with the risk of coronary artery disease (CAD). To date, studies of the 9p21.3 variants on CAD risk mainly focus on the non-coding region of *ANRIL*. However, the biological significance of the variants on *ANRIL* promoter and exons is still unknown. Here we investigate whether the variants on *ANRIL* promoter and exons have an effect on myocardial infarction (MI) risk, and further analyze the association of these variants with the expression of *ANRIL* transcript. We did not find any common variants with minor allele frequencies (MAF) larger than 5% in *ANRIL* promoter by sequencing 1.6kb upstream of the start codon. Unconditional logistic regression analysis revealed that two SNPs in *ANRIL* exons, rs10965215 and rs10738605, were significantly associated with MI risk. Further studies revealed that *ANRIL* transcript *EU741058.1* expression levels of rs10965215 and rs10738605 risk genotypes were borderline lower than those of protective genotypes. Our data provide the evidence that the variants rs10965215 and rs10738605 in *ANRIL* exons contribute to MI risk in the Chinese Han population which might be correlated with the expression of its transcript *EU741058.1*.

## INTRODUCTION

Myocardial infarction (MI) is a major cause of morbidity and mortality worldwide and in China [1, 2], which is ascribed to the combination of environmental and genetic factors. Increasing evidences have revealed that genetic polymorphisms in candidate genes are associated with the risk of MI [3–6].

It is generally accepted that chromosome 9p21.3 is a risk locus for coronary artery disease (CAD) [7–9]. This genomic interval spans 58 kb containing a gene for a long non-coding RNA (lncRNA) known as *ANRIL* (antisense non-coding RNA in the *INK4* locus) [4, 6–8, 10]. *ANRIL* overlaps at its 5′ end with *CDKN2B*, and may have a role in the regulation of expression of adjacent protein coding genes, including *MTAP*, *CDKN2A* (*p15INK4b*) and *CDKN2B* (*p16INK4a*) [4, 6–11]. *ANRIL* alters expression of these associated protein coding genes through multiple mechanisms, including RNA interference, gene silencing, chromatin remodeling, or DNA methylation [12]. Since *CDKN2A* and *CDNK2B* code for two cyclin-dependent kinase inhibitors that play an important role in regulation of the cell cycle and may be implicated in the pathogenesis of atherosclerosis [13], *ANRIL* may be involved in the atherosclerotic process such as in thrombogenesis, vascular remodeling and/or repair, and plaque stability through altering the expression of *CDKN2A* and *CDNK2B*. In addition, *ANRIL* is expressed in endothelial cells, smooth muscle cells, and inflammatory cells known to be stimulated by atherosclerosis and consists of 20 exons subjected to alternative splicing [10, 11, 14, 15]. *ANRIL* expression has been associated with atherosclerosis severity and with CAD-risk genotypes [14, 16], pointing out the importance of *ANRIL* in the mechanism mediating the 9p21.3 association. Moreover, the level of *ANRIL* was significantly decreased in peripheral blood after MI [17]. Above all, *ANRIL* may be a possible candidate gene of CAD and its subcomponent MI at the 9p21.3 risk locus.

Within the 9p21.3 locus, multiple single nucleotide polymorphisms (SNPs) have been showed associated with CAD risk [8, 18, 19]. It has been documented that the risk genotype of rs1333049, located in 3′UTR of *ANRIL*, was associated with reduced expression of *p16INK4a*, *p15INK4b* and *ANRIL*, and with increased VSMC proliferation [20]. Another study demonstrated that subjects homozygous for the risk alleles exhibited increased expression levels of *ANRIL* short transcripts and decreased long variants expression levels, in comparison with those carrying two copies of reference alleles [9]. These results indicated that 9p21.3 variation has an impact on *ANRIL* expression, which in turn influences the expression of the genes involved in the cellular proliferation pathways.

Generally, polymorphisms in lncRNA promoter region may change itself transcriptional activity and thus alter its expression level [21]. Meanwhile, SNPs in exons may directly influence lncRNA structure and affect its stability and expression [21]. Therefore, variants on lncRNA promoter and exon regions deserve our attention in studying disease susceptibility. To date, studies of the 9p21.3 variants on CAD risk mainly focus on the non-coding region of *ANRIL*, such as SNP rs1333049, rs10757274, rs2383206 and so on [9, 20, 22]. However, the effect of variants in *ANRIL* promoter and exon regions on MI risk is still unknown. Therefore, the present study attempted to investigate the associations of the variants on *ANRIL* promoter and exon regions with MI risk in the Chinese Han population. Analyses were performed in a case-control study consisting of 932 subjects (286 MI patients and 646 controls). We also detected the association of the variants with the expression levels of *ANRIL* transcript in peripheral blood mononuclear cells (PBMC) available from MI patients and control subjects.

## RESULTS

### Characteristics of the study population

The clinical characteristics of the studies cohorts were presented in Table 1. There was no statistically significant difference between the MI cases and controls in terms of age. Traditional MI risk factors were prevalent, as we previously reported [3]. In the comparison of lipid profiles, serum triglycerides (TG), total cholesterol (TC), low density lipoprotein cholesterol (LDLC) were higher in the patients than in the controls (*P* < 0.001, *P* = 0.226, *P* < 0.001, respectively), whereas serum high density lipoprotein cholesterol (HDLC) levels were significantly higher among controls (*P* < 0.001). The average fasting plasma glucose (FPG) of the MI cases were significantly higher than that of the controls (*P* < 0.001). MI cases had higher levels of systolic blood pressure, diastolic blood pressure. There was also a higher prevalence of smokers, alcohol consumers, and individuals with hypertension, diabetes or hyperlipidemia among the patients. In addition, the number of female subjects in the cases was much lower than the male subjects. These data demonstrated that male gender, smoking, alcohol consumption, hypertension, hyperlipidemia and diabetes mellitus were the important risk factors for developing MI in the Chinese Han population.

**Table 1:** The characteristics of MI cases and controls

Variable	Controls (*n* = 646)	Cases (*n* = 286)	*P* ^a^
Age (years)	61.58 ± 12.28	62.03 ± 12.04	0.598
Sex (male)	378 (58.5%)	222 (77.6%)	**<0.001**^b^
Smoking	167 (25.9%)	173 (60.5%)	**<0.001**
Drinking	91 (14.1%)	79 (27.6%)	**<0.001**
Hypertension	228 (35.3%)	181 (63.3%)	**<0.001**
Diabetes	105 (16.3%)	135 (47.2%)	**<0.001**
Hyperlipidemia	243 (37.6%)	203 (71.0%)	**<0.001**
Systolic BP (mm Hg)	132.34 ± 18.81	140.21 ± 19.11	**<0.001**
Diastolic BP (mm Hg)	72.82 ± 10.41	75.71 ± 11.56	**<0.001**
FPG (mM)	5.82 ± 1.91	6.61 ± 1.73	**<0.001**
TG (mM)	1.49 ± 0.82	2.05 ± 0.95	**<0.001**
TC (mM)	4.61 ± 1.14	4.70 ± 1.21	0.266
HDLC (mM)	1.37 ± 0.67	1.19 ± 0.39	**<0.001**
LDLC (mM)	2.64 ± 0.91	3.02 ± 0.96	**<0.001**

Abbreviations: BP, blood pressure; FPG, fasting plasma glucose; TG, triglyceride; TC, total cholesterol; HDLC, high density lipoprotein cholesterol; LDLC, low density lipoprotein cholesterol.

^a^ Two-sided chi-square test or independent-samples *t*-test.

^b^ *P* values under 0.05 were indicated in bold font.

### No common variants are detected in *ANRIL* promoter in the Chinese Han population

In this study, the variants in the promoter of *ANRIL* were screened by sequencing 1.6kb upstream of the start codon in MI patients. Our data revealed that there were no common variants in *ANRIL* promoter with minor allele frequencies (MAF) larger than 5% in the Chinese Han population. The distributions of the sequence variants upstream the first exon of *ANRIL* were summarized in Table 2 and Supplementary Figure S1.

**Table 2:** Sequence variants upstream the first base of *ANRIL* start codon in MI patients

Sequence variants	Location ^a^ (bp)	Alleles (*n*)	Genotypes (*n*)	MAF (%)
g.21993325 C > A (rs372433325)	-1465	A (3)	AC (3)	1.5
		C (191)	CC (94)	
g.21993634 C > A (rs192633385)	-1156	A (5)	AC (5)	2.6
		C (189)	CC (92)	
g.21994283 G > A	-507	A (1)	AG (1)	0.5
		G (181)	GG (90)	

Abbreviations: MI, myocardial infarction; MAF, minor allele frequency.

^a^ Locations of variants upstream (-) to the start codon.

### The variants rs10965215 and rs10738605 in *ANRIL* exons confer increased MI risk

Five variants (rs10965215, rs76521274, rs76184305, rs10738605 and rs78766516) located in *ANRIL* exons and whose MAF are larger than 5% in the Chinese Han population were genotyped in 286 MI patients and 646 control subjects. The primary information for these variants was shown in Supplementary Table S1. The observed genotype frequencies of these variants were in Hardy-Weinberg equilibrium among the controls (all *P* values ≥ 0.05, Supplementary Table S1), providing no evidence of population stratification within the dataset.

The allele and genotype distributions of these variants in the MI cases and the controls were shown in Table 3. From the allelic association analysis, we found rs10965215 and rs10738605 showed statistical significance in additive model. The G allele frequency of rs10965215 and C allele frequency of rs10738605 in the MI patients were significantly higher than that in the control group (Table 3). For rs10965215, unconditional logistic regression analysis revealed that G allele had increased MI risk with odds ratio (OR) of 1.37 (95% CI = 1.05-1.78, *P* = 0.020) after adjustment for conventional risk factors compared to A allele. There was a similar trend of the association in dominant model, the combined AG/GG genotypes was associated with the increased MI risk (OR = 1.45, 95% CI = 1.04-2.03, *P* = 0.030) compared to the AA genotype. For rs10738605, C allele conferred increased MI risk with OR of 1.38 (95% CI = 1.06-1.80, *P* = 0.019) compared to G allele after adjustment for conventional risk factors. Similarly, the combined CG/CC genotypes was also associated with the increased MI risk (OR = 1.58, 95% CI = 1.13-2.20, *P* = 0.008) compared to the GG genotype in dominant model. In addition, these two polymorphisms did not show any linkage disequilibrium with the reported MI-associated SNPs (e.g., rs1333049, rs10757274, rs2383206, *etc*.) within *ANRIL* in this study. Taken together, our data indicated that two SNPs in *ANRIL* exons, rs10965215 and rs10738605 were associated with MI risk; rs10965215 G allele and rs10738605 C allele increased individual genetic susceptibility to MI. However, we did not detect any association between rs76521274, rs76184305 or rs78766516 and the risk of MI in allelic or genotypic analyses (Table 3).

**Table 3:** Multivariate associations of the SNPs in *ANRIL* exons with the risk of MI

Type		Controls (*n*= 646)	Cases (*n*= 286)	OR (95% CI) ^a^	*P* ^a^
		No. (%)	No. (%)		
*rs10965215*					
Additive	A	921 (71.3)	386 (67.5)	1.00	-
	G	371 (28.7)	186 (32.5)	1.37 (1.05-1.78)	**0.020** ^b^
Genotype	AA	319 (49.4)	127 (44.4)	1.00	-
	AG	283 (43.8)	132 (46.2)	1.40 (0.99-1.97)	0.061
	GG	44 (6.8)	27 (9.4)	1.81 (0.97-3.40)	0.064
Dominant	AA	319 (49.4)	127 (44.4)	1.00	-
	AG+GG	327 (50.6)	159 (55.6)	1.45 (1.04-2.03)	**0.030**
Recessive	AG+AA	602 (93.2)	259 (90.6)	1.00	-
	GG	44 (6.8)	27 (9.4)	1.53 (0.84-2.80)	0.164
*rs76521274*					
Additive	C	197 (15.2)	88 (15.4)	1.00	-
	T	1095 (84.8)	484 (84.6)	1.07 (0.77-1.49)	0.682
Genotype	CC	11 (1.7)	6 (2.1)	1.00	-
	TC	175 (27.1)	76 (26.6)	1.23 (0.33-4.62)	0.764
	TT	460 (71.2)	204 (71.3)	1.29 (0.35-4.76)	0.701
Dominant	CT+CC	186 (28.8)	82 (28.7)	1.00	-
	TT	460 (71.2)	204 (71.3)	1.07 (0.74-1.53)	0.730
Recessive	CC	11 (1.7)	6 (2.1)	1.00	-
	CT+TT	635 (98.3)	280 (97.9)	1.27 (0.35-4.67)	0.717
*rs76184305*					
Additive	T	191 (14.8)	87 (15.2)	1.00	-
	C	1101 (85.2)	485 (84.8)	0.98 (0.70-1.38)	0.916
Genotype	TT	10 (1.5)	6 (2.1)	1.00	-
	CT	171 (26.5)	75 (26.2)	0.81 (0.20-3.25)	0.768
	CC	465 (72.0)	205 (71.7)	0.85 (0.22-3.32)	0.813
Dominant	CT+TT	181 (28.0)	81 (28.3)	1.00	-
	CC	465 (72.0)	205 (71.7)	1.03 (0.72-1.49)	0.857
Recessive	CT+CC	636 (98.5)	280 (97.9)	1.00	-
	TT	10 (1.0)	6 (2.1)	1.19 (0.31-4.66)	0.798
*rs10738605*					
Additive	G	943 (73.0)	396 (69.2)	1.00	-
	C	349 (27.0)	176 (30.8)	1.38 (1.06-1.80)	**0.019**
Genotype	GG	337 (52.2)	131 (45.8)	1.00	-
	GC	269 (41.6)	134 (46.9)	1.59 (1.13-2.26)	**0.008**
	CC	40 (6.2)	21 (7.3)	1.47 (0.75-2.88)	0.266
Dominant	GG	337 (52.2)	131 (45.8)	1.00	-
	GC+CC	309 (47.8)	155 (54.2)	1.58 (1.13-2.20)	**0.008**
Recessive	GC+GG	606 (93.8)	265 (92.7)	1.00	-
	CC	40 (6.2)	21 (7.3)	1.17 (0.61-2.24)	0.635
*rs78766516*					
Additive	G	1103 (85.4)	500 (87.4)	1.00	-
	A	189 (14.6)	72 (12.6)	0.82 (0.57-1.16)	0.261
Genotype	GG	467 (72.3)	217 (75.9)	1.00	-
	AG	169 (26.2)	66 (23.1)	0.83 (0.57-1.23)	0.354
	AA	10 (1.5)	3 (1.0)	0.56 (0.12-2.65)	0.466
Dominant	AG+GG	636 (98.5)	283 (99.0)	1.00	-
	AA	10 (1.5)	3 (1.0)	0.59 (0.13-2.78)	0.503
Recessive	GG	467 (72.3)	217 (75.9)	1.00	-
	AG+AA	179 (27.7)	69 (24.1)	0.82 (0.56-1.20)	0.299

Abbreviations: SNP, single nucleotide polymorphism; MI, myocardial infarction; OR, odds ratio; CI, confidence interval.

^a^ Adjusted for age, sex, smoking, drinking, hypertension, diabetes, hyperlipidemia.

^b^ *P* values under 0.05 were indicated in bold font.

We further evaluated the genotypes of rs10965215 and rs10738605 and MI susceptibility after stratifying the subjects by age, sex, status of smoking or drinking. As shown in Table 4, we found that the association of rs10965215 and rs10738605 with increased risk in dominant model was more pronounced in females, smokers and non-drinkers. However, no more evident association of rs10965215 and rs10738605 with MI risk was observed among subgroups by age (Table 4).

**Table 4:** Multivariate associations of rs10965215 and rs10738605 polymorphisms with the risk of MI by further stratification for age, sex, smoking and drinking status

		< = 60 years old (*n*= 427)		> 60 years old (*n* = 505)	
		OR (95% CI)	*P* ^a^	OR (95% CI)	*P* ^a^
rs10965215	AA	1.00	-	1.00	-
	AG+GG	1.43 (0.84-2.41)	0.188	1.43 (0.92-2.21)	0.114
rs10738605	GG	1.00	-	1.00	-
	GC+CC	1.49 (0.88-2.52)	0.135	1.58 (1.02-2.46)	0.041
		Male (n=600)		Female (n=332)	
		OR (95% CI)	*P*^b^	OR (95% CI)	*P* ^b^
rs10965215	AA	1.00	-	1.00	-
	AG+GG	1.27 (0.84-1.91)	0.257	2.20 (1.17-4.15)	**0.015**
rs10738605	GG	1.00	-	1.00	-
	GC+CC	1.42 (0.94-2.14)	0.098	2.30 (1.22-4.32)	**0.010**
		Smokers (n=340)		Non-smokers (n=592)	
		OR (95% CI)	*P* ^c^	OR (95% CI)	*P*^c^
rs10965215	AA	1.00	-	1.00	-
	AG+GG	1.74 (1.02-2.95)	**0.041**	1.34 (0.86-2.11)	0.198
rs10738605	GG	1.00	-	1.00	-
	GC+CC	1.72 (1.02-2.91)	**0.042**	1.56 (1.00-2.45)	0.052
		Drinkers (n=170)		Non-drinkers (n=762)	
		OR (95% CI)	*P* ^d^	OR (95% CI)	*P*^d^
rs10965215	AA	1.00	-	1.00	-
	AG+GG	1.82 (0.75-4.46)	0.188	1.46 (1.00-2.12)	**0.049**
rs10738605	GG	1.00	-	1.00	-
	GC+CC	1.40 (0.58-3.34)	0.454	1.68 (1.16-2.45)	**0.007**

Abbreviations: OR, odds ratio; CI, confidence interval.

^a^ Adjusted for sex, smoking, drinking, hypertension, diabetes, hyperlipidemia.

^b^Adjusted for age, smoking, drinking, hypertension, diabetes, hyperlipidemia.

^c^ Adjusted for age, sex, drinking, hypertension, diabetes, hyperlipidemia.

^d^ Adjusted for age, sex, smoking, hypertension, diabetes, hyperlipidemia.

### Association between the haplotypes of *ANRIL* variants and the risk of MI

**Figure 1:**
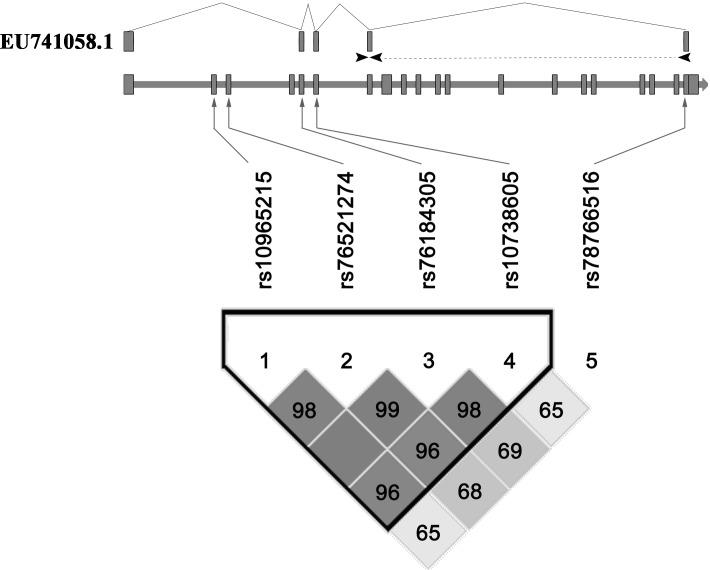
Pairwise linkage disequilibrium between *ANRIL* variants *ANRIL* gene is composed of 20 exons which are represented as boxes. D’ values are plotted as a graph to show linkage disequilibrium between these variants. The schematic of primer binding sites for *EU741058.1* was shown at the top. Arrows represented forward and reverse primers and the reverse primer spanning exons 7 to 20.

Linkage disequilibrium (LD) analysis for the five variants was performed using the Haploview platform [23], and showed that four variants (rs10965215, rs76521274, rs76184305 and rs10738605) were in linkage disequilibrium with D’ ranging from 0.96 to 0.99, indicating they were located in one haplotypic block (Figure 1). Thus we further compared the haplotype frequencies of the four variants between MI group and controls. Four common haplotypes (frequency > 3%) derived from the four variants accounted for almost 100% of the haplotype variations. Among the four common haplotypes, only haplotype rs10965215A-rs76521274T-rs76184305C-rs10738605G was found to have marginal association with decreased MI risk (OR = 0.83, 95% CI = 0.67-1.02, *P* = 0.080, Table 5). When further stratification for drinking was performed, the decreased risk of MI was more evident among non-drinkers with this ATCG haplotype (OR = 0.77, 95% CI = 0.60-0.98, *P* = 0.034, Table 5).

**Table 5:** The association of haplotypes of the SNPs in ANRIL gene with the risk of MI

Haplotype^a^	Controls	Cases	OR (95% CI)	*P*
	No. (%)	No. (%)		
Total	*n* =646	*n* = 286		
A T C G	910.21 (70.5)	380.85 (66.6)	0.83 (0.67-1.02)	0.080
G C T C	188.74 (14.6)	87.00 (15.2)	1.05 (0.79-1.38)	0.749
G T C C	152.65 (11.8)	82.85 (14.5)	1.26 (0.95-1.68)	0.113
G T C G	23.96 (1.9)	15.15 (2.6)	1.44 (0.75-2.76)	0.273
Non-drinkers	*n* =555	*n* = 207		
A T C G	789.61 (71.1)	271.90 (65.7)	0.77 (0.60-0.98)	**0.034**^b^
G C T C	154.74 (13.9)	66.00 (15.9)	1.17 (0.86-1.60)	0.328
G T C C	132.33 (11.9)	62.91 (15.2)	1.32 (0.96-1.83)	0.090
G T C G	20.91 (1.9)	9.10 (2.2)	1.17 (0.53-2.57)	0.697

Abbreviations: SNP, single nucleotide polymorphism; MI, myocardial infarction, OR, odds ratio; CI, confidence interval.

a The allelic sequence in the haplotypes is in the following order: rs10965215, rs76521274, rs76184305, rs10738605.

^b^ *P* values under 0.05 were indicated in bold font.

### Association of *ANRIL* rs10965215 and rs10738605 polymorphisms with its expression

**Figure 2:**
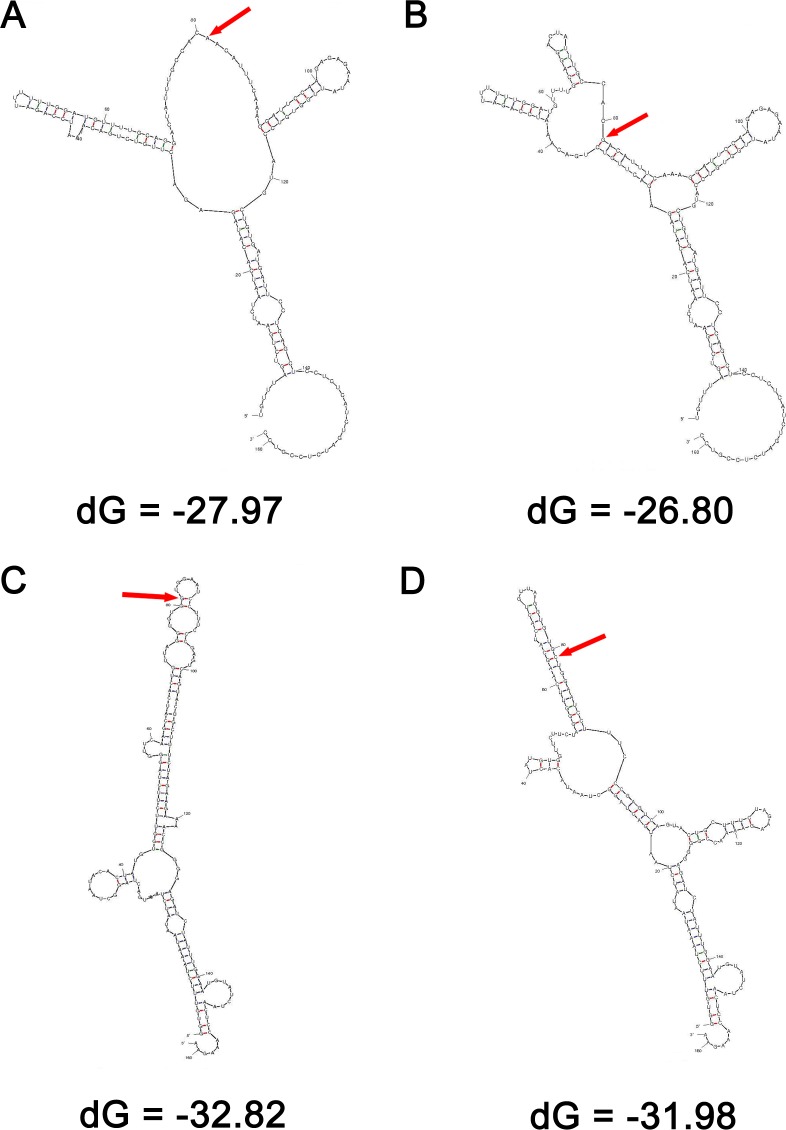
Prediction of the secondary structure of *ANRIL* by Mfold dG, the predicted folding energy. The polymorphism site was indicated by red arrow. The RNA with rs10965215 A allele required lower free energy for folding (dG) compared with the one with rs10965215 G allele (**A**. and **B.)** at 37˚C. The RNA with rs10738605 G allele required lower dG compared with the one with rs10738605 C allele (**C**. and **D.)**

To test whether rs10965215 and rs10738605 may change the secondary structure of *ANRIL*, Mfold analysis was performed. As shown in Figure 2, the RNA with rs10965215 A allele or rs10738605 G allele required lower free energy for folding (dG) compared with the ones with rs10965215 G allele or rs10738605 C allele (−27.97 *versus* −26.80 kcal/mol; −32.82 *versus* −31.98 kcal/mol) at 37˚C. Consistently, these polymorphisms were predicted to result in an obvious change in the secondary structure of *ANRIL*, indicating that these polymorphisms may influence the stability and in turn the expression level of *ANRIL*. To verify this hypothesis, we conducted a correlation analysis between the genotypes and the expression of *ANRIL* transcript *EU741058.1* measured by real-time quantitative RT-PCR.

**Figure 3:**
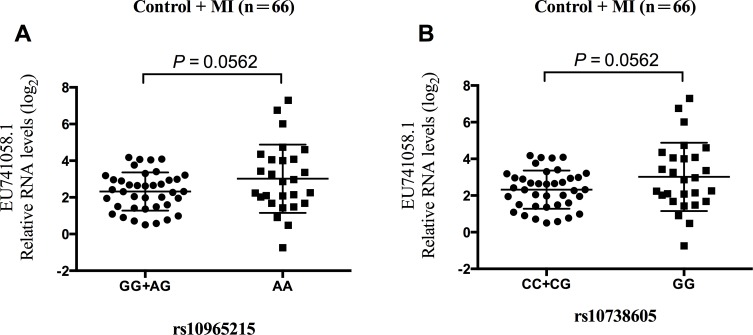
Relationship of *ANRIL* transcript *EU741058.1* with rs10965215 and rs10738605 in PBMCs in the sum of MI patients and control subjects Analysis of *ANRIL* transcript *EU741058.1* expression levels in PBMCs of individuals carrying GG/AG genotypes *vs*. AA genotype for rs10965215 (**A.)** Analysis of *ANRIL* transcript *EU741058.1* expression levels in PBMCs of individuals carrying CC/CG genotypes *vs*. CC genotype for rs10738605 (**B.)**

Our data revealed that the trend were similarly in the association of genotypes with the expression of *ANRIL* transcript *EU741058.1* for both rs10965215 and rs10738605 in the MI patient and control subjects, respectively (Supplementary Figure S2 and Figure S3). *EU741058.1* expression levels of rs10965215 and rs10738605 risk genotypes were lower than those of protective genotypes, though the difference did not reach statistical significance (Supplementary Figure S2 and Figure S3). However, when the MI and control subjects were combined, we observed a borderline statistically significant association of rs10965215 GG/AG genotype with lower expression of *EU741058.1* (*P* = 0.0562, Figure 3A). Similarly, a marginal significant association between the rs10738605 CC/CG genotype and lower expression of *EU741058.1* were observed (*P* = 0.0562, Figure 3B).

## DISCUSSION

MI is a complex disease which is influenced by combined effects of environmental and inherited genetic factors. Genome-wide association studies (GWAS) and many replication studies have demonstrated that variation at the 9p21.3 locus is a major genetic determinant for CAD pathogenesis [4, 6–8, 10, 19, 24]. In addition, GWAS have identified the strongest associations with CAD and other atherosclerotic diseases [4, 7, 25]. However, not all variants in the 3′ end, promoter and exon regions of *ANRIL* have been mapped. Since genetic variants located on these functional regions that influence gene expression or secondary structure are widespread in the human genome, and are responsible for most of the inter-individual variability of normal phenotypes, and also for complex and polygenic diseases [26–28]. We herein aimed to determine whether there were any significant variants in the promoter and exon regions of *ANRIL* or not. We found that there were no common SNPs (MAF > 5%) in the promoter of *ANRIL* by sequencing 1.6kb upstream of the start codon. Our data indicated that two variations in *ANRIL* exons, rs10965215 and rs10738605, which did not show any linkage disequilibrium with the reported MI-associated SNPs, contribute to MI susceptibility in this study. Furthermore, we observed a borderline statistically significant association of rs10965215 and rs10738605 risk genotypes with lower expression of *EU741058.1* than those of protective genotypes.

In 2007, several GWAS consistently identified a region on chromosome 9p21.3 as the most strongly associated with CAD [4, 7, 8, 18]. This finding has been replicated in multiple case-control studies in several population groups in numerous ethnicities following [10, 25, 29–35], making 9p21.3 the most replicated molecular genetic association with CAD to date. This locus spans 58 kb containing a gene for a long non-coding RNA named antisense noncoding RNA in the *INK4* locus (*ANRIL*, also known as *CDKN2BAS*). *ANRIL* overlaps at its 5′ end with *CDKN2B*, and may have a role in the regulation of the expression of adjacent protein coding genes, including *MTAP*, *CDKN2A* and *CDKN2B*, suggesting that SNPs in *ANRIL* are more likely to contribute to the susceptibility of CAD. *ANRIL* is expressed in cells that play a critical role in atherogenesis, such as endothelial cells, vascular smooth muscle cells, and macrophages. Targeted deletion of the orthologous *ANRIL* risk interval in mice can reduce expression of *CDKN2A* and *CDKN2B* in the heart and lead to excessive proliferation of vascular cells [36]. Indeed, subsequent studies showed that *ANRIL* expression is associated with the risk for coronary atherosclerosis, carotid arteriosclerosis, peripheral artery disease, and other vascular diseases [14, 16, 37, 38]. Carriers of the risk alleles showed increased whole blood RNA levels of *ANRIL* short variants *DQ485454* and *EU741058.1*, whereas the long variant *DQ485453* was decreased [9]. Then Holdt *et al*. confirmed the up-regulation of the transcript *EU741058.1*, which was significantly increased in PBMCs and atherosclerotic plaques in carriers of the risk haplotype, but transcript *DQ485454* remains unaffected in this study [14]. They also demonstrated that expressions of the transcripts *EU741058.1* and *NR_003529* were further correlated with the severity of atherosclerosis [14]. Inconsistent with these results, we observed a borderline statistically significant association of rs10965215 and rs10738605 risk genotypes with lower expression of *EU741058.1* than those of protective genotypes. Possible reason for divergent results might be attributable to the use of samples from individuals with clinically suspected CAD as opposed to the combination of healthy individuals and MI patients. Another reason might be that the primers used in the real-time quantitative RT-PCR were isoform-specific in this study. Considering the role of *ANRIL* in cardiovascular diseases, we postulate that risk alleles of rs10965215 and rs10738605 in *ANRIL* exons contribute to MI susceptibility in the Chinese Han population which might be correlated with the expression of its transcript *EU741058.1*.

Genetic variants located on the functional regions, such as promoter and exon regions, could influence gene expression or RNA secondary structure [21]. Since rs10965215 and rs10738605 polymorphisms located in ANRIL exons, we postulated that these two SNPs might affect ANRIL RNA secondary structure, altering its stability and in turn ANRIL expression. In this study, the RNA with rs10965215 A allele or rs10738605 G allele required lower free energy for folding (dG) compared with the ones with rs10965215 G allele or rs10738605 C allele, resulting in an obvious change in the secondary structure of *ANRIL* and indicating that the RNA with rs10965215 A allele or rs10738605 G allele were more stable than the ones with rs10965215 G allele or rs10738605 C allele. Consistent with these results, we observed a borderline statistically significant association of rs10965215 and rs10738605 protective genotypes (AA genotype for rs10956125, GG genotype for rs10738605) with higher expression of *EU741058.1* than those of risk genotypes (AG/GG genotypes for rs10956125, CG/CC genotypes for rs10738605), suggesting that rs10965215 and rs10738605 might affect ANRIL RNA expression probably through altering its secondary structure and stability. Further studies are required to uncover the precise molecular mechanisms of rs10965215 and rs10738605 on the stability and expression of ANRIL.

We are aware that our study had several potential limitations. First, not all variants in *ANRIL* gene were assessed in this study, so complete sequencing will be necessary for systematic identification of potentially causative mutations. Second, we observed a borderline statistically significant association of rs10965215 and rs10738605 risk genotypes with lower expression of *EU741058.1* than those of protective genotypes, these results need to be validated in a larger number of samples or atherosclerotic plaques of MI patients. In addition, replication of this association by independent genetic studies with a larger sample size will be required to confirm our genetic findings.

In summary, we genetically analyzed the variations in promoter and exons of *ANRIL* in a Chinese Han population. Our finding suggests that these two polymorphisms rs10965215 and rs10738605 within *ANRIL* exons contribute to MI risk in the Chinese Han population, although further investigations are required to determine the potential mechanisms by which these polymorphisms influence the MI predisposition.

## MATERIALS AND METHODS

### Study subjects

A total of 286 MI patients and 646 control subjects were recruited from the First People's Hospital of Foshan (Foshan, China) and the Affiliated Hospital of Guangdong Medical University (Zhanjiang, China) from March 2011 to February 2014. All the MI patients were newly diagnosed and previously untreated. Inclusion and exclusion criteria, diagnosis and evaluation as well as criteria for MI were also described previously [3]. Briefly, the diagnosis of MI was based on clinical symptoms and typical electrocardiographic changes, and on increases in the serum cardiac markers, such as creatinine kinase, aspartate aminotransferase, lactate dehydrogenase and troponin T. The diagnosis was confirmed by the identification of the responsible stenosis in any of the major coronary arteries or in the left main trunk by coronary angiography. The control subjects were consecutively recruited from the participating hospitals for regular physical examinations during the same period when MI patients were recruited. The unaffected controls were judged to be free of MI by questionnaires, medical history, clinical examination and electrocardiography. Individuals with congestive heart failure, peripheral vascular disease, rheumatic heart disease, pulmonary heart disease, chronic kidney, hepatic disease, or any malignancy were excluded from the study.

All study subjects were genetically unrelated and self-reported ethnically Han Chinese. Each subject was interviewed after written informed consent was obtained, and a structured questionnaire was administered by interviewers at the enrollment to collect information on demographic data and risk factors related to MI. Meanwhile, we consulted each subject for the genetic relatedness information and excluded the subjects related to the individuals who had enrolled the study. The diagnosis of hypertension was established if patients were on anti-hypertensive medication or if the mean of 3 measurements of systolic blood pressure (SBP) ≥140 mm Hg or diastolic blood pressure (DBP) ≥ 90 mm Hg, respectively. Diabetes mellitus was defined as fasting blood glucose ≥7.0 mmol/L or use of antidiabetic drug therapy. Hyperlipidemia was defined as serum total cholesterol (TC) concentration > 5.72 mmol/L or triglyceride (TG) concentration > 1.70 mmol/L or use of lipid-lowering therapy. Individuals that smoked once a day for over 1 year were defined as smokers. The study was approved by the Medical Ethics Committee of the First People's Hospital of Foshan and the Affiliated Hospital of Guangdong Medical University. All experimental methods applied in this study were carried out according to approved guidelines.

### Analysis of biochemical parameters

An approximately 2 ml venous blood sample was drawn from each subject into tubes containing EDTA after an overnight fast. The blood sample was centrifuged at 2000×g for 15 min immediately after collection and stored at -80°C until analysis. The levels of plasma total cholesterol (TC), triglyceride (TG), high density lipoprotein cholesterol (HDLC), and low density lipoprotein cholesterol (LDLC) were measured enzymatically using a chemistry analyzer (Olympus, Japan). Glucose was analyzed by the glucose oxidase method with an Abbott V/P Analyzer (Abbott Laboratories, USA).

### DNA extraction

Genomic DNA was extracted from peripheral whole blood by TIANamp blood DNA extraction kit (TianGen Biotech, Beijing, China) according to the manufacturer's instructions. All DNA samples were dissolved in water and stored at -20°C until use.

### Sequencing and genotyping

In the preliminary work, we submitted the 1.6kb sequence upstream of the start codon to the Promoter 2.0 to recognize sequence features, and the data showed that this sequence is highly likely prediction promoter with a score of 1.063 [39]. On this basis, the variants in this sequence were screened by sequencing, and our data revealed that there were no common variants in *ANRIL* promoter with minor allele frequencies (MAF) larger than 5% in the Chinese Han population. Finally, five variants (rs10965215, rs76521274, rs76184305, rs10738605 and rs78766516) located in *ANRIL* exons were screened out for genotyping (Supplementary Table S1). These variants cover all the polymorphisms located in *ANRIL* exon regions whose MAF are larger than 5% in the Chinese Han population (Supplementary Table S1). Genomic DNA was genotyped by polymerase chain reaction-ligase detection reaction (PCR-LDR) method (Shanghai Biowing Applied Biotechnology Company). About 5% of the samples were randomly selected to perform the repeated assays and the results were 100% concordant. The sequence of primers and probes are summarized in Supplementary Table S2.

### RNA isolation

Total RNA was extracted from peripheral blood mononuclear cells (PBMCs) of 22 MI patients and 44 control subjects using Trizol (Invitrogen, Carlsbad, CA, USA) according to the manufacturer's instructions. Inclusion criteria for the MI patients were the same as the MI cases enrolled in the study. Quality and quantity of the RNAs were assessed by A260/A280 nm reading using NanoDrop1000 spectrophotometer (NanoDrop Technologies, Wilmington, DE, USA). RNA integrity was determined by running an aliquot of the RNA samples on a denaturing agarose gel stained with SYBR Green I.

### Real-time quantitative RT-PCR

SYBR green-based quantitative real-time polymerase chain reaction was used to examine the change in expression level of *ANRIL* transcript *EU741058.1* in RNAs prepared from PBMCs of 22 MI patients and 44 control subjects. The isoform-specific primers used for real-time quantitative RT-PCR were provided in Supplementary Table S3 and the primer binding sites for *EU741058.1* were shown in Figure 1. With too little blood from each subject, it is not enough to perform independent RNA isolation from the same sample. Therefore, we represent the technical replicates of a single RNA isolation. The relative mRNA expression levels were normalized to the housekeeping gene *ACTIN* and presented as log2-transormed expression (ΔΔCt).

### Prediction of *ANRIL* secondary structure

Mfold (version 3.2) was applied to evaluate the effect of these polymorphisms on *ANRIL* (GenBank: NT_008413.17) secondary structure. Mfold is an internet-based RNA folding program (http://unafold.rna.albany.edu/?q = mfold/RNA-Folding-Form) used for the prediction of mRNA secondary structure [40]. The 161-bp *ANRIL* mRNA fragments including corresponding polymorphism sites and flanking sequences (80 bp each in the 3 and 5 flanking regions) were analyzed and the free energy required for folding (dG) was calculated, respectively.

### Statistical analysis

The statistical power analysis was performed using PS program (Power and Sample size calculations, Version 3.0.43) [41]. All the variants were tested for confirmation with Hardy-Weinberg expectations by a goodness-of-fit χ2 test among the control subjects. Quantitative variables were expressed as mean ± standard deviation (SD), and qualitative variables were expressed as percentages. The differences of the demographic characteristics between the cases and controls were estimated using the χ2 test (for categorical variables) and student's *t* test (for continuous variables). Association between the variants and the risk for MI was evaluated using logistic regression analysis, adjusted by age, sex, smoking, drinking, hypertension, diabetes and hyperlipidemia. The statistical analyses were performed using the SPSS software (version 21). The haplotype analysis on the polymorphisms was done using the SHEsis platform freely available online (http://analysis.bio-x.cn/myAnalysis.php) [42]. Statistical differences of *ANRIL* expression levels between different groups of samples in real-time RT-PCR experiment were determined by Mann-Whitney U-test. *P* < 0.05 was used as the criterion of statistical significance.

## SUPPLEMENTARY MATERIALS FIGURES AND TABLES



## Abbreviations

ANRIL: antisense non-coding RNA in the INK4 locus
CAD: coronary artery disease
MI: myocardial infarction
PCR-LDR: polymerase chain reaction-ligase detection reaction
PBMC: peripheral blood mononuclear cell
OR: odds ratio
CI: confidence interval
CDKN2B: cyclin-dependent kinase inhibitor 2B
CDKN2A: cyclin-dependent kinase inhibitor 2A
MTAP: methyl-thioadenosine phosphorylase
SNP: single nucleotide polymorphism
VSMC: vascular smooth muscle cell
SBP: systolic blood pressure
DBP: diastolic blood pressure
TC: total cholesterol
TG: triglyceride
HDLC: high density lipoprotein cholesterol
LDLC: low density lipoprotein cholesterol
FPG: fasting plasma glucose
MAF: minor allele frequencies
SD: standard deviation
LD: Linkage disequilibrium
GWAS: Genome-wide association studies
