# Supplementary material for: Variants in ANRIL gene correlated with its expression contribute to myocardial infarction risk
Source: Oncotarget. 2017 Jan 18;8(8):12607–19. doi: 10.18632/oncotarget.14721 (PMC5355039; doi:10.18632/oncotarget.14721)
Supplement: Supplementary file 1 [file oncotarget-08-12607-s001.pdf]

Variants in *ANRIL* gene correlated with its expression contribute to myocardial infarction risk

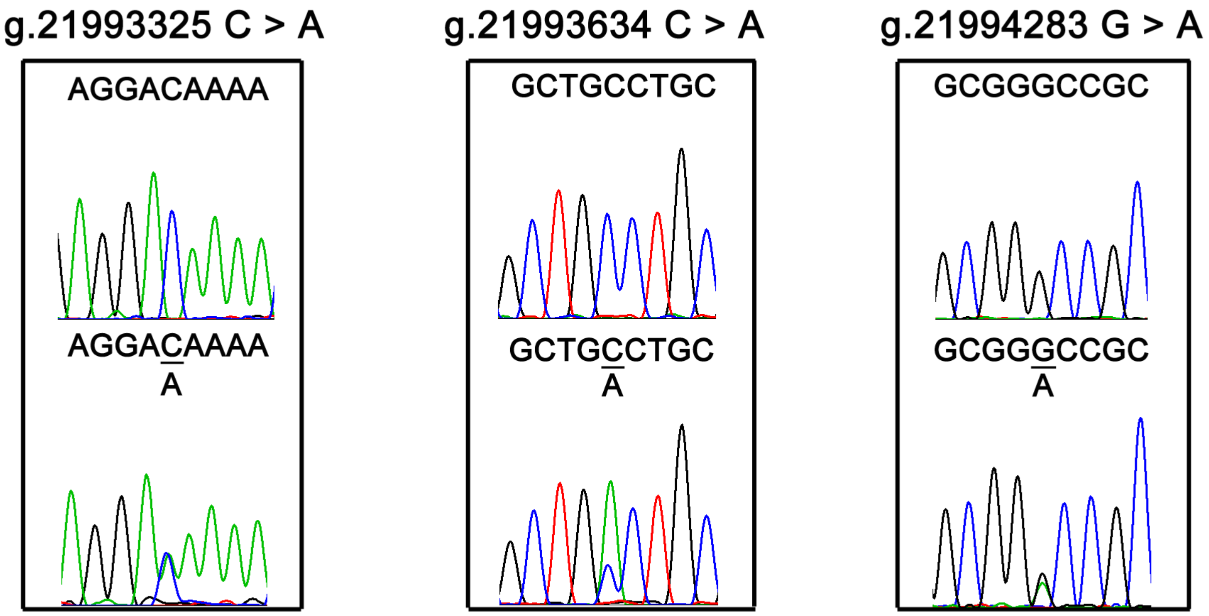

Supplementary Figure S1: The sequence variants identified upstream ANRIL start codon in 100 MI patients. The polymorphic sites are underlined.

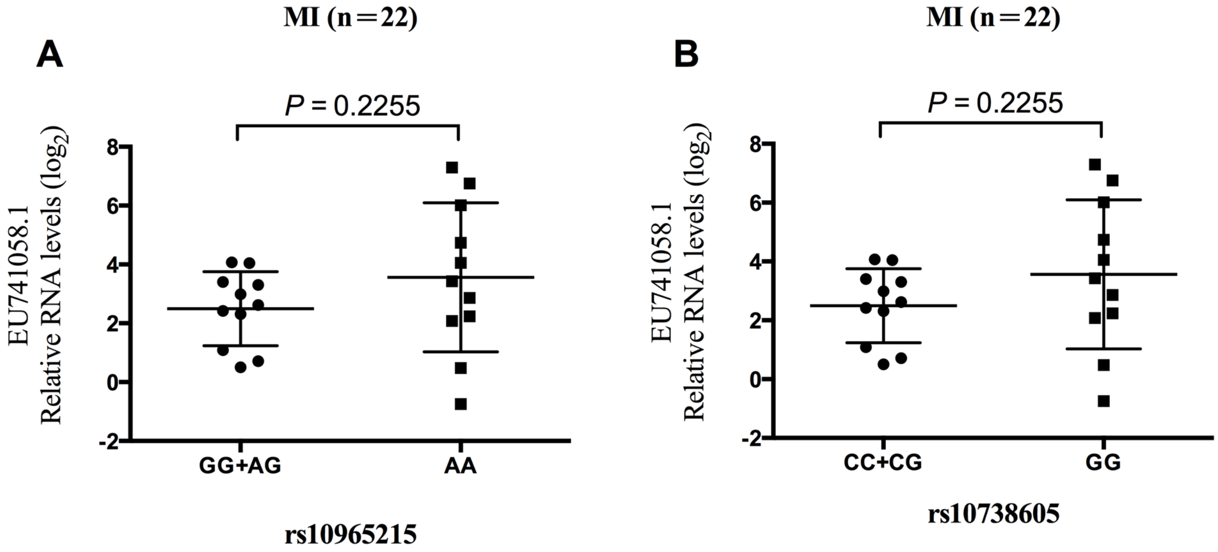

Supplementary Figure S2: Relationship of ANRIL transcript EU741058.1 with rs10965215 and rs10738605 in PBMCs in MI patients. Analysis of *ANRIL* transcript *EU741058.1* expression levels in PBMCs of MI patients carrying GG/AG genotypes vs. AA genotype for rs10965215 (A). Analysis of *ANRIL* transcript *EU741058.1* expression levels in PBMCs of MI patients carrying CC/CG genotypes vs. CC genotype for rs10738605 (B).

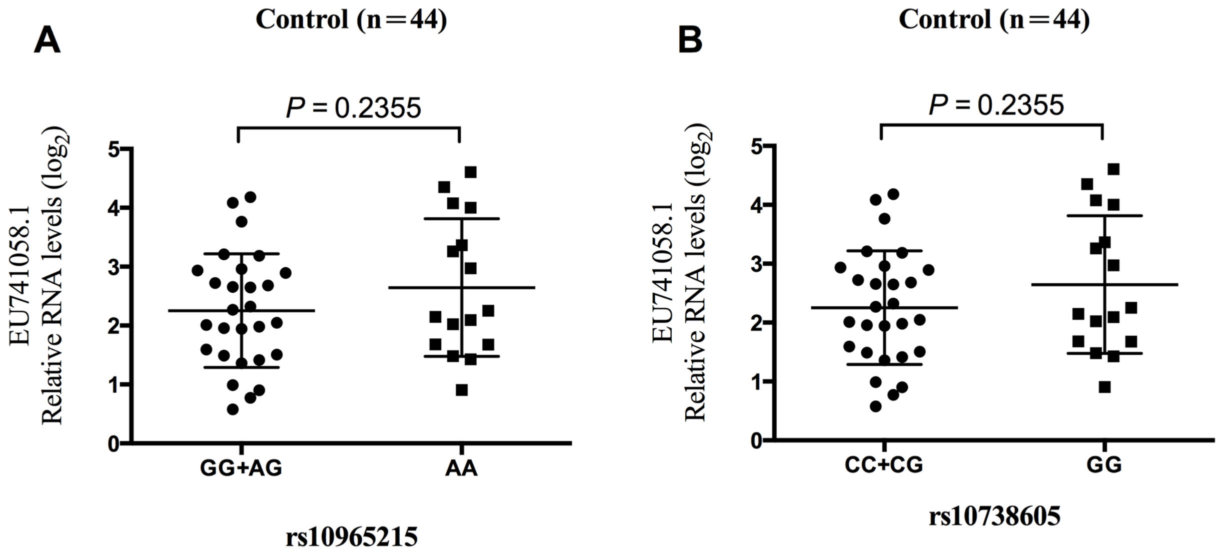

Supplementary Figure S3: Relationship of ANRIL transcript EU741058.1 with rs10965215 and rs10738605 in PBMCs in control subjects. Analysis of *ANRIL* transcript *EU741058.1* expression levels in PBMCs of control subjects carrying GG/AG genotypes vs. AA genotype for rs10965215 (A). Analysis of *ANRIL* transcript *EU741058.1* expression levels in PBMCs of control subjects carrying CC/CG genotypes vs. CC genotype for rs10738605 (B).
